# Tri­carbonyl­chlorido­(6′,7′-di­hydro-5′*H*-spiro­[cyclo­hexane-1,6′-dipyrido[3,2-*d*:2′,3′-*f*][1,3]diazepine]-κ^2^ *N* ^1^,*N* ^11^)rhenium(I)

**DOI:** 10.1107/S1600536813024288

**Published:** 2013-09-04

**Authors:** Oliver R. Clegg, Lindsay P. Harding, John W. Miller, Craig R. Rice

**Affiliations:** a Department of Chemical & Biological Sciences, University of Huddersfield, Queensgate, Huddersfield HD1 3DH, England

## Abstract

In the title compound, [ReCl(C_16_H_18_N_4_)(CO)_3_], the Re^I^ ion is coordinated in a distorted octa­hedral geometry by one Cl atom, two N atoms of the bidentate ligand and three carbonyl groups. The cyclo­hexane group is orientated in a *transoid* fashion with respect to the chloride ligand. In the crystal, N—H⋯Cl hydrogen bonds link complex mol­ecules, forming a two-dimensional network parallel to (100).

## Related literature

For a review of the photophysical properties of Re–polypyridyl complexes, see: Coleman *et al.* (2008 ▶). For the synthesis of [Re(3,3′-di­amino-2,2′-bi­pyridine)(CO)_3_Cl] and for the preparation of oxo-steroid derivatives of [Re(3,3′-di­amino-2,2′-bi­pyridine)(CO)_3_Cl], see: Bullock *et al.* (2012 ▶). For the reaction of [Re(3,3′-di­amino-2,2′-bi­pyridine)(CO)_3_Cl] with ketones, see: Clayton *et al.* (2008 ▶). For the structure of the cyclo­pentane analogue of the title compound, see: Clegg *et al.* (2013 ▶).
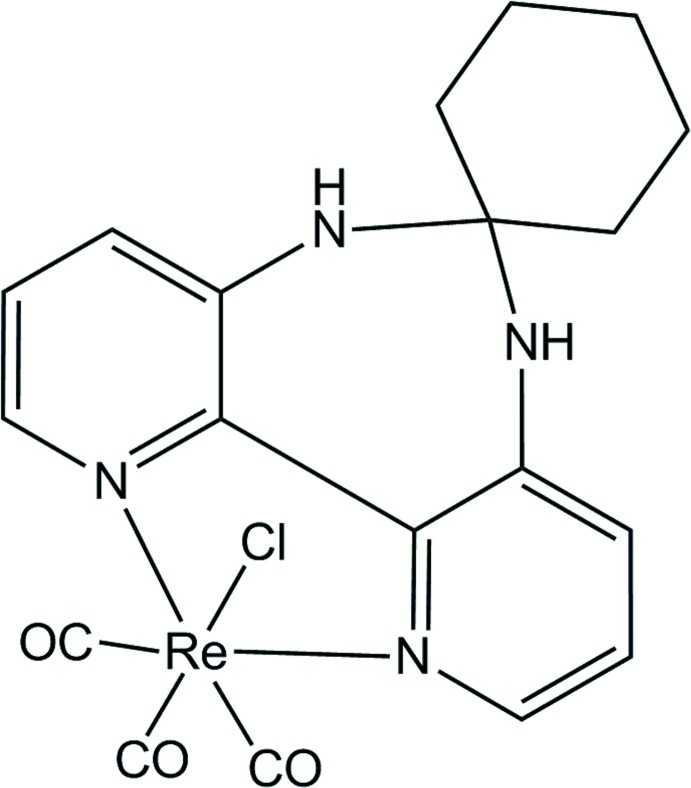


## Experimental

### 

#### Crystal data

[ReCl(C_16_H_18_N_4_)(CO)_3_]
*M* *_r_* = 572.02
Monoclinic, 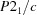

*a* = 12.6794 (6) Å
*b* = 11.9040 (6) Å
*c* = 12.7732 (6) Å
β = 97.066 (1)°
*V* = 1913.29 (16) Å^3^
*Z* = 4
Mo *K*α radiation
μ = 6.52 mm^−1^
*T* = 150 K
0.10 × 0.10 × 0.03 mm

#### Data collection

Bruker APEXII CCD diffractometer
Absorption correction: multi-scan (*SADABS*; Bruker, 2009 ▶) *T*_min_ = 0.562, *T*_max_ = 0.828
22579 measured reflections
5590 independent reflections
4543 reflections with *I* > 2σ(*I*)
*R*_int_ = 0.039

#### Refinement

*R*[*F* ^2^ > 2σ(*F* ^2^)] = 0.027
*wR*(*F* ^2^) = 0.051
*S* = 1.04
5590 reflections
259 parameters
2 restraints
H atoms treated by a mixture of independent and constrained refinement
Δρ_max_ = 1.08 e Å^−3^
Δρ_min_ = −0.74 e Å^−3^

### 

Data collection: *APEX2* (Bruker, 2009 ▶); cell refinement: *SAINT* (Bruker, 2009 ▶); data reduction: *SAINT*; program(s) used to solve structure: *SHELXS97* (Sheldrick, 2008 ▶); program(s) used to refine structure: *SHELXL97* (Sheldrick, 2008 ▶); molecular graphics: *OLEX2* (Dolomanov *et al.*, 2009 ▶); software used to prepare material for publication: *OLEX2*.

## Supplementary Material

Crystal structure: contains datablock(s) I, New_Global_Publ_Block. DOI: 10.1107/S1600536813024288/lh5645sup1.cif

Structure factors: contains datablock(s) I. DOI: 10.1107/S1600536813024288/lh5645Isup2.hkl

Additional supplementary materials:  crystallographic information; 3D view; checkCIF report

## Figures and Tables

**Table 1:** Hydrogen-bond geometry (Å, °)

*D*—H⋯*A*	*D*—H	H⋯*A*	*D*⋯*A*	*D*—H⋯*A*
N3—H3⋯Cl1^i^	0.89 (3)	2.64 (2)	3.417 (3)	146 (3)
N4—H4⋯Cl1^ii^	0.89 (3)	2.46 (3)	3.334 (3)	171 (3)

Symmetry codes: (i) 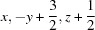; (ii) 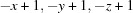.

**Fig. 1.**
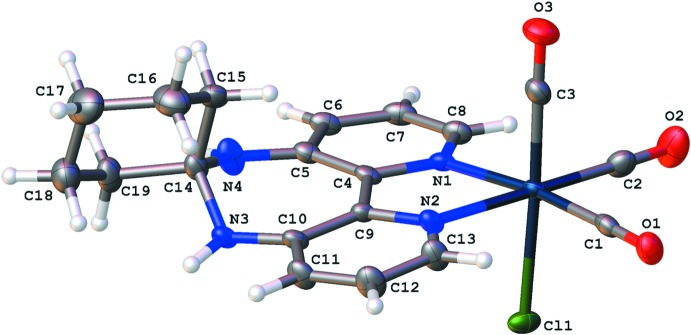
The molecular structure of the title compound with displacement ellipsoids shown at the 50% probability level.

## supplementary crystallographic information

### 1. Comment

The title complex was prepared as part of a larger study into conjugation of [Re(3,3'-diamino-2,2'-bipyridine)(CO)_3_Cl] with oxo-steroids to form luminescent derivatives (Bullock *et al.* 2012). These steroids contain a cyclohexyl ring (ring A) with a ketone group in the 3-position; therefore, cyclohexanone was used as a model compound to examine the potential reactivity of such steroids with the rhenium complex.

Single-crystal X-ray analysis of the title complex gave the structure shown in Fig. 1. The rhenium centre adopts a distorted octahedral geometry which is coordinated by two nitrogen atoms from a 3,3'-diamino-2,2'-bipyridyl ligand and two carbonyl ligands in the equatorial sites and by a carbonyl ligand and a chloride ion in the axial sites. The cyclohexyl ring adopts a chair conformation and is orientated in a *trans*-oid fashion relative to the chloride ion on the rhenium centre. In the crystal, N—H···Cl hydrogen bonds (Table 1 & Fig. 2) link complex molecules to form a two-dimensional network parallel (100).

A similar compound has been prepared using cyclopentanone instead of cyclohexanone. The title compound compound is essentially isostructural with that compound (Clegg *et al.* 2013).

### 2. Experimental

To a solution of [Re(3,3'-diamino-2,2'-bipyridine)(CO)_3_Cl] in dichloromethane was added cyclohexanone (10 µ*L*, *ca*. 2 eq.) and a few grains of camphorsulfonic acid. The solution was stirred at room temperature for 2 h. The resulting precipitate was filtered *in vacuo*, washed with dichloromethane and dried, giving the product as a yellow solid. Crystals suitable for X-ray analysis were prepared by slow evaporation of an acetonitrile solution of the complex.

### 3. Refinement

All non-hydrogen atoms were refined anisotropically. Hydrogen atoms on *sp*^2^ and *sp*^3^ C atoms were placed in calculated positions and refined with riding constraints and isotropic displacement parameters 1.2 × their parent carbon atoms. H atoms bonded to N atoms were refined independently with a bond length constraint of 0.91 (2)Å and U_iso_(H) = 1.2U_eq_(N).

### Crystal data

**Table:**

[ReCl(C_16_H_18_N_4_)(CO)_3_]	*F*(000) = 1104
*M**_r_* = 572.02	*D*_x_ = 1.986 Mg m^−^^3^
Monoclinic, *P*2_1_/*c*	Mo *K*α radiation, λ = 0.71073 Å
Hall symbol: -P 2ybc	Cell parameters from 6672 reflections
*a* = 12.6794 (6) Å	θ = 2.4–30.2°
*b* = 11.9040 (6) Å	µ = 6.52 mm^−^^1^
*c* = 12.7732 (6) Å	*T* = 150 K
β = 97.066 (1)°	Block, yellow
*V* = 1913.29 (16) Å^3^	0.10 × 0.10 × 0.03 mm
*Z* = 4	

### Data collection

**Table:**

Bruker APEXII CCD diffractometer	5590 independent reflections
Radiation source: fine-focus sealed tube	4543 reflections with *I* > 2σ(*I*)
Graphite monochromator	*R*_int_ = 0.039
φ and ω scans	θ_max_ = 30.0°, θ_min_ = 2.4°
Absorption correction: multi-scan (*SADABS*; Bruker, 2009)	*h* = −17→17
*T*_min_ = 0.562, *T*_max_ = 0.828	*k* = −16→16
22579 measured reflections	*l* = −17→17

### Refinement

**Table:**

Refinement on *F*^2^	Primary atom site location: structure-invariant direct methods
Least-squares matrix: full	Secondary atom site location: difference Fourier map
*R*[*F*^2^ > 2σ(*F*^2^)] = 0.027	Hydrogen site location: inferred from neighbouring sites
*wR*(*F*^2^) = 0.051	H atoms treated by a mixture of independent and constrained refinement
*S* = 1.04	*w* = 1/[σ^2^(*F*_o_^2^) + (0.0164*P*)^2^ + 1.9168*P*] where *P* = (*F*_o_^2^ + 2*F*_c_^2^)/3
5590 reflections	(Δ/σ)_max_ = 0.002
259 parameters	Δρ_max_ = 1.08 e Å^−^^3^
2 restraints	Δρ_min_ = −0.74 e Å^−^^3^

### Special details

**Table:**

Geometry. All e.s.d.'s (except the e.s.d. in the dihedral angle between two l.s. planes) are estimated using the full covariance matrix. The cell e.s.d.'s are taken into account individually in the estimation of e.s.d.'s in distances, angles and torsion angles; correlations between e.s.d.'s in cell parameters are only used when they are defined by crystal symmetry. An approximate (isotropic) treatment of cell e.s.d.'s is used for estimating e.s.d.'s involving l.s. planes.
Refinement. Refinement of *F*^2^ against ALL reflections. The weighted *R*-factor *wR* and goodness of fit *S* are based on *F*^2^, conventional *R*-factors *R* are based on *F*, with *F* set to zero for negative *F*^2^. The threshold expression of *F*^2^ > σ(*F*^2^) is used only for calculating *R*-factors(gt) *etc*. and is not relevant to the choice of reflections for refinement. *R*-factors based on *F*^2^ are statistically about twice as large as those based on *F*, and *R*- factors based on ALL data will be even larger.

### Fractional atomic coordinates and isotropic or equivalent isotropic displacement parameters (Å^2^)

**Table:**

	*x*	*y*	*z*	*U*_iso_*/*U*_eq_	
Re1	0.209524 (11)	0.453951 (10)	0.250974 (9)	0.01842 (4)	
Cl1	0.33970 (7)	0.60427 (7)	0.21693 (7)	0.02644 (18)	
N1	0.3302 (2)	0.4016 (2)	0.37600 (19)	0.0185 (5)	
N2	0.1863 (2)	0.5567 (2)	0.38525 (19)	0.0199 (5)	
N3	0.3158 (2)	0.6174 (2)	0.6529 (2)	0.0215 (6)	
N4	0.4188 (2)	0.4503 (3)	0.6590 (2)	0.0310 (7)	
O1	0.0385 (2)	0.5624 (2)	0.09147 (18)	0.0306 (6)	
O2	0.2669 (3)	0.3038 (2)	0.0706 (2)	0.0477 (8)	
O3	0.0489 (2)	0.2781 (2)	0.3017 (2)	0.0387 (7)	
C1	0.1030 (3)	0.5197 (3)	0.1503 (2)	0.0226 (7)	
C2	0.2461 (3)	0.3609 (3)	0.1381 (3)	0.0286 (8)	
C3	0.1102 (3)	0.3443 (3)	0.2834 (2)	0.0255 (7)	
C4	0.3337 (2)	0.4527 (3)	0.4731 (2)	0.0189 (6)	
C5	0.4093 (3)	0.4144 (3)	0.5566 (2)	0.0209 (7)	
C6	0.4833 (3)	0.3319 (3)	0.5338 (3)	0.0227 (7)	
H6	0.5368	0.3080	0.5878	0.027*	
C7	0.4792 (3)	0.2859 (3)	0.4357 (3)	0.0255 (7)	
H7	0.5293	0.2307	0.4206	0.031*	
C8	0.4001 (3)	0.3219 (3)	0.3585 (3)	0.0229 (7)	
H8	0.3954	0.2887	0.2905	0.027*	
C9	0.2552 (2)	0.5444 (3)	0.4760 (2)	0.0175 (6)	
C10	0.2467 (3)	0.6191 (3)	0.5607 (2)	0.0195 (6)	
C11	0.1681 (3)	0.7017 (3)	0.5486 (3)	0.0268 (8)	
H11	0.1617	0.7523	0.6050	0.032*	
C12	0.1001 (3)	0.7113 (3)	0.4572 (3)	0.0309 (8)	
H12	0.0465	0.7675	0.4495	0.037*	
C13	0.1117 (3)	0.6367 (3)	0.3764 (3)	0.0273 (8)	
H13	0.0651	0.6425	0.3125	0.033*	
C14	0.3416 (3)	0.5122 (3)	0.7096 (2)	0.0234 (7)	
C15	0.2419 (3)	0.4398 (3)	0.7144 (3)	0.0296 (8)	
H15A	0.2073	0.4253	0.6419	0.036*	
H15B	0.2628	0.3665	0.7474	0.036*	
C16	0.1653 (4)	0.4967 (4)	0.7762 (3)	0.0432 (10)	
H16A	0.1025	0.4480	0.7792	0.052*	
H16B	0.1411	0.5678	0.7410	0.052*	
C17	0.2176 (4)	0.5216 (4)	0.8886 (3)	0.0514 (13)	
H17A	0.1662	0.5610	0.9281	0.062*	
H17B	0.2374	0.4500	0.9254	0.062*	
C18	0.3166 (4)	0.5941 (4)	0.8870 (3)	0.0401 (10)	
H18A	0.2956	0.6691	0.8580	0.048*	
H18B	0.3516	0.6044	0.9600	0.048*	
C19	0.3935 (3)	0.5414 (3)	0.8214 (3)	0.0335 (8)	
H19A	0.4228	0.4720	0.8567	0.040*	
H19B	0.4534	0.5937	0.8165	0.040*	
H3	0.305 (3)	0.678 (2)	0.691 (3)	0.040*	
H4	0.479 (2)	0.433 (3)	0.699 (3)	0.040*	

### Atomic displacement parameters (Å^2^)

**Table:**

	*U*^11^	*U*^22^	*U*^33^	*U*^12^	*U*^13^	*U*^23^
Re1	0.01938 (6)	0.01328 (6)	0.02209 (6)	0.00027 (6)	0.00050 (4)	−0.00139 (5)
Cl1	0.0213 (4)	0.0212 (4)	0.0355 (4)	−0.0005 (3)	−0.0016 (3)	0.0099 (3)
N1	0.0202 (14)	0.0109 (13)	0.0243 (12)	−0.0002 (11)	0.0020 (11)	0.0016 (10)
N2	0.0188 (13)	0.0153 (13)	0.0243 (12)	0.0019 (12)	−0.0032 (10)	−0.0022 (10)
N3	0.0240 (15)	0.0139 (14)	0.0251 (13)	−0.0009 (12)	−0.0037 (12)	−0.0009 (10)
N4	0.0241 (16)	0.0336 (17)	0.0322 (14)	0.0131 (15)	−0.0091 (12)	−0.0073 (14)
O1	0.0313 (14)	0.0297 (14)	0.0285 (12)	0.0044 (12)	−0.0052 (11)	−0.0035 (10)
O2	0.073 (2)	0.0393 (17)	0.0327 (14)	0.0243 (16)	0.0140 (15)	−0.0042 (12)
O3	0.0384 (17)	0.0295 (15)	0.0479 (15)	−0.0119 (13)	0.0033 (13)	−0.0002 (12)
C1	0.0260 (18)	0.0169 (17)	0.0252 (15)	−0.0050 (14)	0.0042 (14)	−0.0069 (12)
C2	0.035 (2)	0.0219 (18)	0.0287 (16)	0.0060 (17)	0.0044 (15)	0.0051 (14)
C3	0.029 (2)	0.0202 (18)	0.0258 (15)	0.0002 (15)	−0.0018 (14)	−0.0042 (13)
C4	0.0164 (14)	0.0124 (14)	0.0272 (14)	−0.0039 (14)	−0.0005 (12)	−0.0013 (12)
C5	0.0183 (16)	0.0157 (15)	0.0281 (15)	−0.0021 (13)	0.0002 (13)	−0.0006 (12)
C6	0.0153 (16)	0.0156 (16)	0.0362 (17)	0.0022 (13)	−0.0008 (14)	0.0016 (12)
C7	0.0216 (18)	0.0154 (16)	0.0402 (18)	0.0034 (14)	0.0072 (15)	0.0018 (13)
C8	0.0234 (18)	0.0146 (16)	0.0308 (16)	0.0033 (14)	0.0045 (14)	−0.0009 (12)
C9	0.0186 (15)	0.0122 (14)	0.0209 (12)	−0.0008 (14)	−0.0013 (11)	0.0017 (12)
C10	0.0196 (16)	0.0116 (15)	0.0262 (14)	−0.0005 (13)	−0.0024 (13)	0.0003 (11)
C11	0.029 (2)	0.0181 (17)	0.0319 (16)	0.0064 (15)	−0.0016 (15)	−0.0077 (13)
C12	0.033 (2)	0.0236 (19)	0.0348 (18)	0.0124 (17)	−0.0033 (16)	−0.0046 (14)
C13	0.0263 (19)	0.0232 (18)	0.0300 (16)	0.0091 (16)	−0.0061 (14)	−0.0033 (14)
C14	0.0254 (18)	0.0201 (17)	0.0227 (14)	0.0045 (14)	−0.0052 (13)	−0.0018 (12)
C15	0.040 (2)	0.0168 (18)	0.0308 (16)	−0.0015 (16)	−0.0001 (16)	0.0025 (13)
C16	0.043 (3)	0.039 (2)	0.049 (2)	−0.009 (2)	0.012 (2)	−0.0029 (19)
C17	0.071 (4)	0.045 (3)	0.042 (2)	−0.001 (2)	0.022 (2)	−0.0035 (19)
C18	0.048 (3)	0.040 (2)	0.0311 (19)	−0.001 (2)	−0.0006 (18)	−0.0039 (16)
C19	0.037 (2)	0.033 (2)	0.0267 (15)	0.0077 (19)	−0.0094 (15)	−0.0033 (15)

### Geometric parameters (Å, º)

**Table:**

Re1—C3	1.895 (4)	C7—H7	0.9500
Re1—C1	1.914 (3)	C8—H8	0.9500
Re1—C2	1.919 (4)	C9—C10	1.414 (4)
Re1—N2	2.156 (3)	C10—C11	1.395 (5)
Re1—N1	2.163 (3)	C11—C12	1.368 (5)
Re1—Cl1	2.5083 (9)	C11—H11	0.9500
N1—C8	1.335 (4)	C12—C13	1.383 (5)
N1—C4	1.377 (4)	C12—H12	0.9500
N2—C13	1.338 (4)	C13—H13	0.9500
N2—C9	1.370 (4)	C14—C19	1.537 (4)
N3—C10	1.378 (4)	C14—C15	1.538 (5)
N3—C14	1.464 (4)	C15—C16	1.488 (6)
N3—H3	0.895 (18)	C15—H15A	0.9900
N4—C5	1.367 (4)	C15—H15B	0.9900
N4—C14	1.440 (5)	C16—C17	1.534 (6)
N4—H4	0.895 (19)	C16—H16A	0.9900
O1—C1	1.158 (4)	C16—H16B	0.9900
O2—C2	1.154 (4)	C17—C18	1.526 (6)
O3—C3	1.151 (4)	C17—H17A	0.9900
C4—C5	1.418 (4)	C17—H17B	0.9900
C4—C9	1.482 (4)	C18—C19	1.500 (5)
C5—C6	1.413 (5)	C18—H18A	0.9900
C6—C7	1.362 (5)	C18—H18B	0.9900
C6—H6	0.9500	C19—H19A	0.9900
C7—C8	1.386 (5)	C19—H19B	0.9900
			
C3—Re1—C1	89.74 (14)	N3—C10—C11	118.4 (3)
C3—Re1—C2	89.72 (15)	N3—C10—C9	122.9 (3)
C1—Re1—C2	86.96 (14)	C11—C10—C9	118.6 (3)
C3—Re1—N2	93.30 (12)	C12—C11—C10	121.3 (3)
C1—Re1—N2	98.55 (11)	C12—C11—H11	119.4
C2—Re1—N2	173.72 (13)	C10—C11—H11	119.4
C3—Re1—N1	93.94 (12)	C11—C12—C13	118.0 (3)
C1—Re1—N1	172.11 (11)	C11—C12—H12	121.0
C2—Re1—N1	100.02 (13)	C13—C12—H12	121.0
N2—Re1—N1	74.30 (10)	N2—C13—C12	122.2 (3)
C3—Re1—Cl1	176.99 (10)	N2—C13—H13	118.9
C1—Re1—Cl1	90.96 (10)	C12—C13—H13	118.9
C2—Re1—Cl1	93.23 (12)	N4—C14—N3	109.8 (3)
N2—Re1—Cl1	83.70 (8)	N4—C14—C19	107.2 (3)
N1—Re1—Cl1	85.01 (7)	N3—C14—C19	108.1 (3)
C8—N1—C4	121.0 (3)	N4—C14—C15	109.8 (3)
C8—N1—Re1	120.3 (2)	N3—C14—C15	111.4 (3)
C4—N1—Re1	118.6 (2)	C19—C14—C15	110.5 (3)
C13—N2—C9	121.2 (3)	C16—C15—C14	111.1 (3)
C13—N2—Re1	120.1 (2)	C16—C15—H15A	109.4
C9—N2—Re1	118.5 (2)	C14—C15—H15A	109.4
C10—N3—C14	121.0 (3)	C16—C15—H15B	109.4
C10—N3—H3	110 (3)	C14—C15—H15B	109.4
C14—N3—H3	117 (3)	H15A—C15—H15B	108.0
C5—N4—C14	127.2 (3)	C15—C16—C17	110.5 (4)
C5—N4—H4	116 (3)	C15—C16—H16A	109.6
C14—N4—H4	116 (3)	C17—C16—H16A	109.6
O1—C1—Re1	177.8 (3)	C15—C16—H16B	109.6
O2—C2—Re1	178.9 (4)	C17—C16—H16B	109.6
O3—C3—Re1	179.0 (3)	H16A—C16—H16B	108.1
N1—C4—C5	118.6 (3)	C18—C17—C16	111.0 (3)
N1—C4—C9	113.6 (3)	C18—C17—H17A	109.4
C5—C4—C9	127.8 (3)	C16—C17—H17A	109.4
N4—C5—C6	115.6 (3)	C18—C17—H17B	109.4
N4—C5—C4	126.2 (3)	C16—C17—H17B	109.4
C6—C5—C4	118.2 (3)	H17A—C17—H17B	108.0
C7—C6—C5	121.2 (3)	C19—C18—C17	111.3 (3)
C7—C6—H6	119.4	C19—C18—H18A	109.4
C5—C6—H6	119.4	C17—C18—H18A	109.4
C6—C7—C8	118.2 (3)	C19—C18—H18B	109.4
C6—C7—H7	120.9	C17—C18—H18B	109.4
C8—C7—H7	120.9	H18A—C18—H18B	108.0
N1—C8—C7	122.5 (3)	C18—C19—C14	112.4 (3)
N1—C8—H8	118.7	C18—C19—H19A	109.1
C7—C8—H8	118.7	C14—C19—H19A	109.1
N2—C9—C10	118.7 (3)	C18—C19—H19B	109.1
N2—C9—C4	114.7 (3)	C14—C19—H19B	109.1
C10—C9—C4	126.6 (3)	H19A—C19—H19B	107.9

### Hydrogen-bond geometry (Å, º)

**Table:**

*D*—H···*A*	*D*—H	H···*A*	*D*···*A*	*D*—H···*A*
N3—H3···Cl1^i^	0.89 (3)	2.64 (2)	3.417 (3)	146 (3)
N4—H4···Cl1^ii^	0.89 (3)	2.46 (3)	3.334 (3)	171 (3)

Symmetry codes: (i) *x*, −*y*+3/2, *z*+1/2; (ii) −*x*+1, −*y*+1, −*z*+1.
